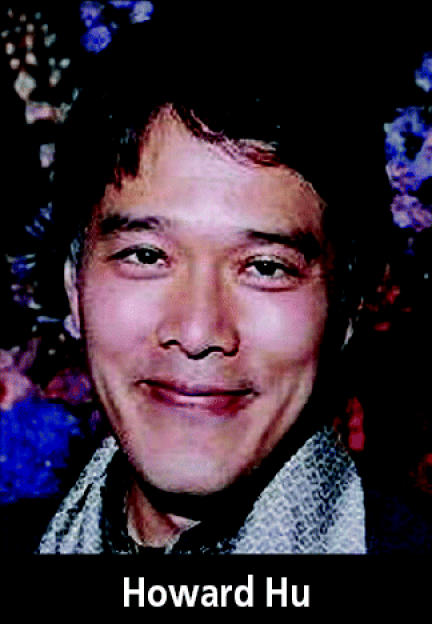# Editorial: The Future of Environmental Medicine in *Environmental Health Perspectives*: Where Should We Be Headed?

**DOI:** 10.1289/ehp.113-1280414

**Published:** 2005-09

**Authors:** Brian S. Schwartz, Gary Rischitelli, Howard Hu

**Affiliations:** Johns Hopkins Bloomberg School of Public Health and School of Medicine, Baltimore, Maryland, E-mail: bschwart@jhsph.edu; Oregon Health Sciences University, Portland, Oregon; Harvard School of Public Health and School of Medicine, Boston, Massachusetts

In 1998, *Environmental Health Perspectives* (*EHP*) began “Grand Rounds in Environmental Medicine” as a regular feature (Hu 1998; Hu and Woolf 2003). This soon led to an expanded Environmental Medicine section that aimed to regularly publish articles in the Grand Rounds format as well as reviews, commentaries, case reports, and research articles, all of relevance to environmental medicine, with a focus mainly on clinical practice. The Grand Rounds series has been a resounding success, as reflected by the wide range of environmental medicine topics, the diversity of reporting sources, and the increased physician readership of the journal. Howard Hu served as the first Medical Editor from 1996 to 2004 and was then succeeded by Brian Schwartz; also in 2004, the journal appointed two Associate Medical Editors: Howard Hu and Gary Rischitelli.

With this change in medical editorship, we wish to take the opportunity to present our views on the scope of the “environment” and “environmental medicine” and what we believe is the expanding nature of the field. We present this as a sounding board for ideas and not in any way as a definitive discussion of these issues. Our overall goal is to increase physician readership of the journal and engage *EHP* ’s audience on a wide variety of environmental medicine topics relevant to both clinical and public health practice; this editorial is a first step in achieving this goal.

We are in a changing world. The world’s population continues to grow and the proportion of the elderly is increasing, with recognition of the susceptibility to health risks not only in early life but also in late life. The United States, once the greatest manufacturing nation in history, has become primarily a service economy. Many of the high-intensity hazardous exposures that were the traditional focus of early environmental and occupational health practitioners and social reformers are not the threat to large U.S. populations that they once were. Some exposures of concern remain as pockets of high-intensity hazards or as widespread chronic low-level hazards; new threats are being identified; and environmental factors and gene–environment interactions are thought to play a greater role in the etiology of many diseases than previously believed (Newton and Schwartz 2005; Schwartz 2005a, 2005b).

We are quickly approaching a global economy, in which there is free movement of capital, goods, information, and services, if not free movement of labor. A great deal of manufacturing has shifted to developing countries, accompanied by levels of hazardous exposures not seen in the developed world in decades (LaDou 1994, 2002, 2003), and these countries frequently have few occupational or environmental health resources to address such hazards (LaDou 2002, 2003). With this global economy, we have witnessed a global movement of exposures and other environmental hazards. Toxicants released in one geographic area can influence health at great distances, and agents not previously considered to be pollutants, such as carbon dioxide, are having profound impacts on global climate patterns. Global environmental health threats now include not only climate change but also ecosystem decay, species loss, deforestation and desertification, sea level rise, fisheries decline, and stratospheric ozone depletion, to name but a few.

Traditionally, environmental health specialists have thought about the ways that the environment can influence human health in terms of the hazardous agents, sources, and routes of exposure. This led to a focus on how human activities have resulted in contamination of air, food, water, and soil, and, in turn, how the contaminants can adversely affect human health. The purview of environmental health has more recently been expanded to bear on the potential impact on health of other components of the environment and on multiple scales, from local to global.

Although the definition of “environment,” as it pertains to environmental health, is not our main focus in this editorial, it has clearly been evolving and needs to be articulated, so its effects on human health and medical practice in relation to each other can be articulated more clearly. To that end, we discuss the environment in terms of four main domains: the natural, anthropogenic, social, and cultural environments.

The natural environment includes those features that did not arise from human activities, and can include radon; earthquakes, volcanism, and other natural disasters; cosmic ionizing radiation; certain infectious diseases; and other similar hazards. The anthropogenic environment is what has been created or altered by humans; it includes hazards from toxicants—the traditional practice core of environmental medicine—as well as the built environment, and how this may influence health-related behaviors. In contrast to declines in manufacturing and traditional hazardous exposures in the United States, land use has been dramatically outstripping population growth in most parts of the country (Frumkin et al. 2004). A growing literature describes how urban sprawl and the local food environment can influence health (Ewing 2005; Ewing et al. 2003a; Ewing et al. 2003b; Handy et al. 2002).

The social environment involves the interactions between people in various places, and can include factors such as social disorganization, safety, physical disorder, commercial vitality, and economic deprivation, measured at the neighborhood level. A growing literature has documented a contextual effect of the social environment on a variety of health outcomes after control for critical individual-level risk factors (Pickett and Pearl 2001); perhaps more important, the social environment may affect several underlying biologic pathways that can modify how the body responds to toxicants or other traditional environmental hazardous exposures (McEwen 2000a, 2000b, 2000c, 2002).

Finally, the cultural environment is important because, for example, cultural norms can influence an individual’s interaction with the environment; if the cultural norm is to engage in physical activity, it is more likely that individual community members will also do so. Without consideration of this influence, for example, studies of land use and physical activity could reach erroneous conclusions.

Clearly, as the concept of “environment” evolves from the traditional notions of hazards to the built, social, and cultural environments on several scales, it has expanded or created new interfaces with the fields of individual and social behavior, psychology, sociology, ecology, land use, architecture, and other disciplines. This suggests to us that the role of physicians in environmental health and medicine must also evolve if they are to remain relevant and for physicians to have an impact in addressing individual and population health threats that can arise from these old and new environmental challenges.

In the face of these trends, and given the evolution of the concept of “environment” as it pertains to health, what, then, is “environmental medicine”? While one primary focus of environmental medicine should continue to include the health concerns of individual patients resulting from hazardous exposures, it should also expand to encompass the larger notion of “environment” and include a particular focus on population health.

In the mainstream scientific literature, environmental medicine is the work of clinicians and has been generally defined as the evaluation, management, and study of detectable human disease or adverse health outcomes from exposure to external physical, chemical, and biologic factors in the general environment [Ducatman 1993; Ducatman et al. 1990; Institute of Medicine (IOM) 1995]. Over a decade ago, the IOM specified competency-based objectives for environmental medicine education (IOM 1995). Although this report and its predecessors (IOM 1990, 1991, 1993) included discussion of epidemiology, population health, and nonmedical interventions, the overwhelming focus was on the clinical assessment and medical management of patients with individual illness due to hazardous exposures (IOM 1995). The tension between the focus on the clinical evaluation of patients and the broader goals of environmental medicine was recognized (IOM 1993).

Unfortunately, education in environmental medicine remains a low priority in U.S. medical schools and postgraduate clinical training programs (Burstein and Levy 1994; Schenk et al. 1996). Moreover, it is clear that the landscape of what can be claimed to be environmental medicine in practice has become complex and diverse. Although its roots are in traditional allopathic clinical practice, many environmental medicine physicians are primarily involved in public health practice and use quantitative and management skills that focus on populations rather than the clinical paradigm that focuses on individuals.

The traditional, allopathic practice of clinical environmental medicine has evolved in developed countries with a steady decline in the need for diagnosis and treatment of disease caused by high-level exposures as these countries have made the epidemiologic transition from short-latency, acute effects of high-intensity doses to long-latency, chronic effects of low-intensity cumulative doses. In the United States, the legal implications of exposures have increased, whereas making causal connections has become more challenging, more contentious, and more often opposed by well-funded industry groups (Michaels 2005).

Perhaps as a result of the aforementioned epidemiologic transition and the failure of allopathic practitioners in caring for patients with nonspecific, symptom-based disorders that the patients believe are caused by low-level environmental exposures, alternative medicine practice for patients with environmental concerns has become increasingly common. Nontraditional use of diagnostic and treatment procedures that have little medical scientific justification for their use has become a regular part of this practice. The scientific community has not shed much light on understanding the causes and optimal management approaches for these conditions; therefore, it is probably no surprise that the management of such patients has become the purview of alternative systems of care. To confuse matters even more, the term “environmental medicine” has been adopted by practitioners of a branch of alternative medicine also known as “clinical ecology.” To date, this practice has not been evidence-based and cannot be considered a validated approach to such patients.

On the other hand, environmental health practitioners long ago showed that we could prevent environmental disease without completely understanding the cause, the specific toxic agent, genetic polymorphisms, or mechanistic pathways. For example, in the 18th century, Sir Percival Pott stopped an epidemic of scrotal cancer in chimney sweeps by asking them to improve their genital hygiene (Pott 1775), while knowing little about the cause, biology or mechanism of this disease. Environmental health specialists involved in public health practice thus have a right to ask, “Must we wait for incontrovertible scientific evidence of health effects and causes in the face of impressive trends in environmental degradation?” If so, how does this assist the goal of protecting the public’s health?

Clinical environmental medicine practice is at a crossroads, especially in the United States. Traditional hazardous exposures that formed the base of practice continue to decline in the United States, while health concerns have become aroused by lower and lower levels of exposures, with many patients often finding their way to non-traditional practice. There are still high-intensity exposures, especially in the developing world and in poor and minority communities in the United States. Although traditional exposures on average are declining in the United States, new concerns do arise, and research and public health practice expertise must be brought to bear on these issues.

We believe that environmental medicine must continue to have a patient-based arm of practice, but it must increasingly encompass newer and broader concerns, especially regarding the health of populations and public health practice. In addition to clinical expertise, population, communication, and policy-change skills must be increasingly used. To assess risks and help effect change, practitioners must possess biomedical, epidemiologic, and management skills, and these practitioners should include physicians involved in environmental medicine.

*EHP* is the ideal vehicle for providing information to the diverse group of physicians practicing in the expanding field of environmental medicine. What are these diverse physician “practices,” and what knowledge and skills do these need? We have identified five categories of “practices”: environmental and occupational medicine (EOM) clinical practice specialists; EOM public health practitioners; international EOM practitioners (in the developing world); community-based primary care providers; and academic EOM physician-scientists.

EOM clinical practice specialists must be able to diagnose and provide medical and nonmedical management of all environmental diseases, translate new research results to practice, and make complex causal inferences. By contrast, EOM public health practitioners have the clinical skills to diagnose environmentally related disease and interpret clinical data, but they are mainly focused on population, not individual health, and are thus interested in policy change and the management of environmental exposures and issues. They are primarily interested in prevention, not diagnosis and treatment, and acknowledge that a case of EOM disease represents a failure of prevention and thus often a failure of EOM policy. Such practitioners embrace the importance of the precautionary principle: while researchers are waiting for the last bit of scientific evidence to be generated, climate is changing, habitat is being destroyed, species are being lost, and disease is being caused. For many environmental health issues, the time to act is now.

In the developing world, international EOM practitioners (LaDou 2003) still face traditional hazards and high-level exposures. Many lack specialty training or access to expertise. They must be able to diagnose and treat common EOM diseases and assess and manage emerging health threats from rapid industrialization.

Primary care providers must be able to recognize sentinel cases in the community—which requires making inferences about cause—and then take appropriate steps, often by getting relevant public health authorities involved, to prevent additional cases. Making inferences about cause requires skills in exposure and dose estimation, and getting public health authorities involved requires knowledge of reporting requirements and responsible parties. Such practitioners also must be able to provide knowledgeable advice to their patients about what is known about new causes; patients want to know what must be done about the myriad environmental concerns that appear in the lay press and other sources of such information. The challenge is how to provide information on these topics to these practitioners in an efficient and easily learned way.

Finally, academic EOM physician/scientists are directing or collaborating in multidisciplinary research that capitalizes on both clinical knowledge in individuals and epidemiologic information from populations and results in new knowledge of clinically translatable value. They work on a wide variety of research topics and use a full range of clinical, epidemiologic, and environmental health science skills to shed light on the environmental exposures and complex causal pathways that underlie the pathogenesis and progression of disease.

We have described the expanding scope of environmental medicine and physician practice in it. As editors we want to be encompassing and not exclusionary about what we believe are appropriate articles for the Environmental Medicine section. We want to continue the highly successful Grand Rounds in Environmental Medicine and encourage continued submissions of such articles, focused on either clinical practice or population-based practice, especially from developing countries. We welcome submissions that evaluate interventions of potential importance in individuals or in populations. Environmental medicine is not just about clinical practice for patients concerned about environmental diseases; thus, we welcome submissions of epidemiologic and community-based studies that have relevance to the public health practice of environmental medicine. We hope our ideas stimulate thought and discussion on these topics and look forward to receiving your submissions.

## Figures and Tables

**Figure:**
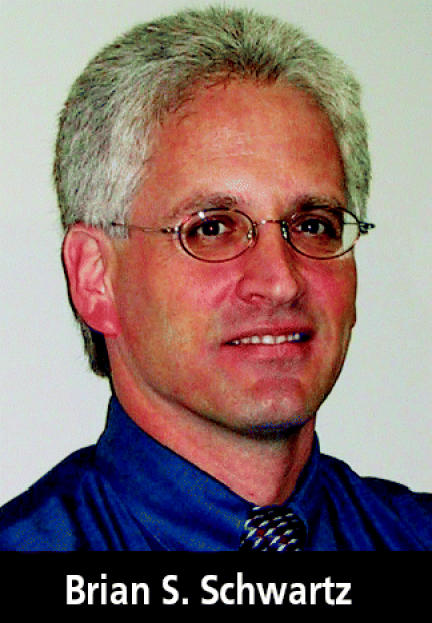


**Figure:**
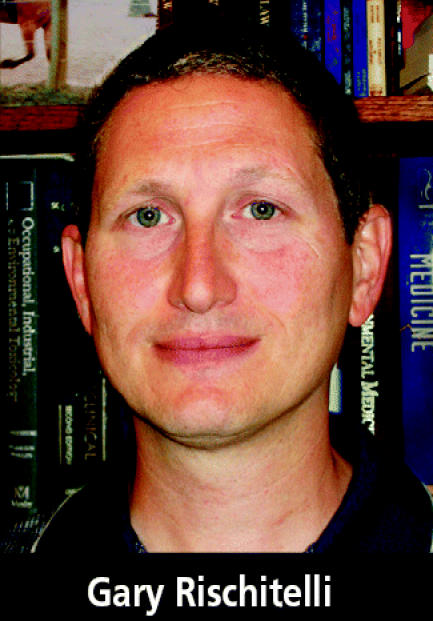


**Figure:**